# Analysis of Bonding Defects in Cementing Casing Using Attenuation Characteristic of Circumferential SH Guided Waves

**DOI:** 10.3390/s26010332

**Published:** 2026-01-04

**Authors:** Jie Gao, Tianhao Chen, Yan Lyu, Guorong Song, Jian Peng, Cunfu He

**Affiliations:** 1 School of Information Science and Technology, Beijing University of Technology, Beijing 100124, China; 2 State Run Luoyang Dancheng Wireless Power Plant, Luoyang 471000, China

**Keywords:** cementing casing, bonding defect, circumferential SH guided wave, dispersion characteristics, attenuation characteristics

## Abstract

**Highlights:**

**What are the main findings?**

- Based on the state matrix and Legendre polynomial hybrid method, a new numerical method is presented for the investigation of the propagation characteristic of C-SH waves in cementing casing.
- The relationship between the amplitude of SH guided waves and the size of the bonding defects is established through the attenuation coefficient.
- The size of the bonding defects can be experimentally predicted by the attenuation distribution curve.

**What are the implications of the main findings?**

- It provides a numerical analysis method and experimental prediction basis for the detection of bonding defects in cemented casing.

**Abstract:**

Circumferential guided wave detection technology can serve as an alternative method for detecting casing bond defects. Due to the presence of the cement cladding, the circumferential SH guided waves transmit shear waves into the cement cladding as they propagate in the cementing casing, which cause the circumferential SH guided waves to show attenuation characteristics. In this study, the cementing casing structure was considered as a steel substratum semi-infinite domain cemented cladding pipe structure, and the corresponding dispersion and attenuation characteristics of circumferential SH guided waves were numerically solved based on the state matrix and Legendre polynomial hybrid method. In addition, a finite element simulation model of cementing casing was established to explore the interaction between SH guided waves and bonding defects. The relationship between the amplitude of SH guided waves and the size of the bonding defects was established through the attenuation coefficient. Moreover, an experimental platform for cementing casing detection is constructed to detect bonding defects of different sizes and to achieve the acoustic analysis of cementing defects in cementing casing, which provides a research path for the non-destructive testing and evaluation of bonding defects in cementing casing.

## 1. Introduction

Cementing is one of the important steps in the process of drilling and completion of wells, through the reinforcement of the well wall, to ensure that the subsequent drilling is carried out successfully and serves the safe production of oil and gas [1]. Due to the complex conditions within the wells, the cement-casing bond surface is prone to defects such as bonding defects, leading to cracks on the inner wall of the casing [2]. This severely compromises the safe extraction of oil and gas resources [3]. Hence, non-destructive testing of cementing quality is particularly crucial for ensuring the safety of oil and gas extraction.

Acoustic logging technology, as a major means of the detection of cementing quality, has gradually evolved from acoustic amplitude logging [4] and acoustic variable density logging [5] to sectoral cement cementing logging [6] and ultrasonic pulse echo logging [7], etc. With the advancement of cementing technology, low-resistance cement has been progressively adopted for cementing operations in deepwater wells and low-pressure, leak-prone zones [8]. However, the low-impedance cement exhibits acoustic impedance values close to those of fluids, and the above acoustic logging methods are mainly based on acoustic impedance logging, which makes it difficult to effectively detect the bonding defects in cementitious surfaces [9]. More reliable methods for cementing quality inspection and evaluation still need to be developed [10].

Ultrasonic guided wave detection technology has the advantages of high detection efficiency, large detection range, and high detection sensitivity, and is widely used in the detection of composite plates, pipes, and other structures [11]. Also, the circumferential guided wave detection technique is commonly used for the detection of pipe defects [12], and circumferential SH waves have attracted significant attention from numerous scholars in fields such as cementing quality assessment. Meanwhile, compared to piezoelectric sensors, electromagnetic sensors enable non-contact measurement and are better positioned to meet the demands of cementing quality inspection in complex downhole environments [13]. Based on electromagnetic acoustic guided wave detection technology, Patterson [14] experimentally analyzed the circumferential SH wave attenuation characteristics of cementing casing with different cement properties. Gong [15] conducted simulation and experimental studies on the curing state of cement media outside casing using electromagnetic ultrasonic SH guided waves. The results indicate that the attenuation characteristics of SH guided waves can be utilized to evaluate the curing state. Rodgers [16] employed circumferential SH0 guided waves to detect axial cracks in steel pipes. By analyzing the residual signal generated through baseline subtraction from the defective reflection signal, non-destructive testing of axial defects in steel pipes can be achieved. However, none of the aforementioned studies have conducted quantitative characterization analyses of the cementing defects in casing.

In addition, it is necessary to clarify the propagation mechanism of ultrasonic guided waves along cement-cemented layers in cementing casings within semi-infinite steel-matrix domains, which is crucial for optimizing parameters and guiding experimental analysis [17]. Matuszyk [18] theoretically analyzed circumferential SH waves in a casing by using the semi-analytical finite element method and the perfectly matched layer method, and the effect of changing the casing thickness and the properties of the cement behind the casing was investigated. At this point, due to the presence of the cement liner within the cementing casing, the circumferential SH guided wave exhibits significant attenuation characteristics, which is also known as the leaky guide wave [19]. Georgiades [20] employed the Chebyshev polynomial spectral configuration method to solve for the dispersion curves and attenuation curves of leaky Lamb waves in an elastic waveguide with a fluid on either side. Hayashi [21] developed a semi-analytical finite element method for calculating leaky Lamb waves in a single side and both two side water-loaded plates. Based on the finite element method, Castaings used absorbing layers and attenuating materials to simulate an infinite solid material for leaky media to solve analytically for guided waves within waveguides at arbitrary interfaces. However, the theoretical research on the propagation characteristics of circumferential guided waves has primarily focused on hollow pipes. There are fewer theoretical studies on the attenuation characteristics of circumferential SH guided waves in cementing casing structures [22]. There is still a need for more reliable and accurate methods for theoretical analyses of the propagation and attenuation characteristic solutions.

Although ultrasonic guided waves show promise, the existing theoretical research has primarily focused on hollow pipes or utilized semi-analytical finite element methods for limited casing scenarios, lacking a rigorous quantitative characterization of attenuation in semi-infinite domain cemented structures. Furthermore, conventional acoustic logging relies heavily on impedance contrast, which struggles to detect defects effectively in low-impedance cement environments. To address these gaps, this study introduces a state matrix and Legendre polynomial hybrid method to accurately model the “steel substratum semi-infinite domain” structure. Unlike previous qualitative studies, we establish a quantitative mathematical relationship between the C-SH wave attenuation coefficient and defect size. This approach not only overcomes the limitations of impedance-based logging but also provides a robust theoretical and experimental framework for predicting the precise dimensions of bonding defects.

In this study, a theoretical numerical approach for analyzing the circumferential SH guided wave propagation behavior in cementing casing is developed, and the circumferential SH guided wave dispersion curves and attenuation curves of the steel substratum semi-infinite domain cemented cladding pipe structure are numerically solved based on the state vector method and Legendre polynomial hybrid method. The interaction between bonding defects and the propagation characteristic of circumferential SH guided waves is explored through finite element simulation and cementing casing detection experiments, and the relationship between the amplitude of SH guided waves and the size of the bonding defects is established through the attenuation coefficient.

## 2. Modeling Analysis of C-SH Guided Wave Characteristics

### 2.1. Theoretical Consideration of C-SH Waves’ Behavior

**Figure 1:**
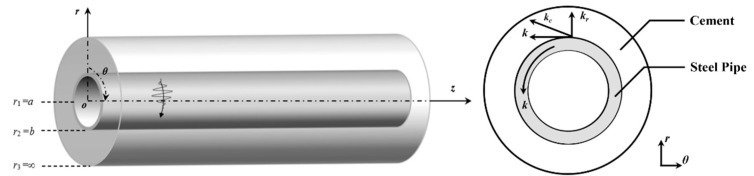
Schematic diagram of multi-layered cementing casing structure.

Considering that the cementing casing structure is a steel substratum semi-infinite domain cement cladding pipe structure, the inner radius of the steel substratum is *a*, and the outer radius is *b*. The cement cladding seems to be a semi-infinite domain medium, as shown in Figure 1. In the cylindrical coordinate system (*r*, *θ*, *z*), the geometric structure extends infinitely in the *z*-direction. The circumferential SH guided wave propagates along the *θ* direction in the substratum, and the wave number vector *k* is in the *θ* direction. When the circumferential SH guided wave propagates in the substratum layer, the transmission occurs at the interface between the substratum and the cladding, and the SH wave in the cladding propagates along the direction of the wave number vector *k_c_*.

To facilitate the theoretical derivation, the following assumptions are explicitly made:

1. Material properties: Both the steel substratum and the cement cladding are isotropic, linear elastic materials.
2. Geometry: The structure is infinitely long in the axial (z) direction, and the cement cladding is treated as a semi-infinite domain.
3. Deformation: The modeling is based on the small deformation assumption.
4. External conditions: Body forces are negligible, and the system is analyzed without external loads other than the guided wave excitation.

Assuming that both the substratum and the semi-infinite domain cladding are isotropic linear elastic materials, the corresponding constitutive relationship can be expressed as follows:

(1) $$\sigma_{\alpha \beta }=C_{\alpha \beta \gamma \upsilon }\varepsilon_{\gamma \upsilon },\;\alpha ,\beta ,\gamma ,\upsilon \in \left\{r,\theta ,z\right\}$$

Based on the small deformation assumption, the strain–displacement relationships in terms of the cylindrical coordinate system are as follows:

(2) $$\begin{array}{l} \varepsilon_{rr}=\frac{\partial u_{r}}{\partial r},\;\varepsilon_{r\theta }=\frac{1}{2}\left(\frac{1}{r}\cdot \frac{\partial u_{r}}{\partial \theta }+\frac{\partial u_{\theta }}{\partial r}-\frac{u_{\theta }}{r}\right)\\ \varepsilon_{\theta \theta }=\frac{1}{r}\cdot \frac{\partial u_{\theta }}{\partial \theta }+\frac{u_{r}}{r},\;\varepsilon_{rz}=\frac{1}{2}\left(\frac{\partial u_{r}}{\partial z}+\frac{\partial u_{z}}{\partial r}\right)\\ \varepsilon_{zz}=\frac{\partial u_{z}}{\partial z},\;\varepsilon_{\theta z}=\frac{1}{2}\left(\frac{\partial u_{\theta }}{\partial z}+\frac{\partial u_{z}}{r\cdot \partial \theta }\right) \end{array}$$

where *u* represents the displacement component, *σ* represents the stress component, *ε* represents the strain component, and *C* is the elastic constant. Based on the theory of elastic mechanics, the wave equations of guided wave without body force are as follows:

(3) $$\begin{array}{l} \frac{\partial \sigma_{rr}}{\partial r}+\frac{1}{r}\frac{\partial \sigma_{r\theta }}{\partial \theta }+\frac{\partial \sigma_{rz}}{\partial z}+\frac{\sigma_{rr}-\sigma_{\theta \theta }}{r}=\rho \frac{\partial^{2}u_{r}}{\partial t^{2}}\\ \frac{\partial \sigma_{\theta r}}{\partial r}+\frac{1}{r}\frac{\partial \sigma_{\theta \theta }}{\partial \theta }+\frac{\partial \sigma_{\theta z}}{\partial z}+\frac{2\sigma_{r\theta }}{r}=\rho \frac{\partial^{2}u_{\theta }}{\partial t^{2}}\\ \frac{\partial \sigma_{rz}\;}{\partial r}+\frac{1}{r}\frac{\partial \sigma_{z\theta }}{\partial \theta }+\frac{\partial \sigma_{zz}}{\partial z}+\frac{\sigma_{rz}}{r}=\rho \frac{\partial^{2}u_{z}}{\partial t^{2}} \end{array}$$

where *ρ* represents the density. To transform the basic equation of the elastic wave into the form of state matrix, the displacement and stress fields need to be written in vector form. Since the SH wave propagates along the *θ* direction, the harmonic propagation factor is defined as $e^{i\left(k\theta -\omega t\right)}$. The form of the displacement and stress state vector is as follows:

(4) $$\begin{array}{l} u={\left[\begin{array}{ccc} u_{r} & u_{\theta } & u_{z} \end{array}\right]}^{T}e^{i(k\theta -\omega t)}\\ \tau_{i}={\left[\begin{array}{ccc} \sigma_{ir} & \sigma_{i\theta } & \sigma_{iz} \end{array}\right]}^{T}e^{i(k\theta -\omega t)} \end{array}$$

where *k* represents the wave number and *ω* represents the angular frequency.

Using the displacement and stress state vectors, the wave equations can be rewritten as follows:

(5) $$\frac{\partial r\tau_{r}}{\partial r}=-r\rho \omega^{2}u-\frac{\partial \tau_{\theta }}{\partial \theta }-r\frac{\partial \tau_{z}}{\partial z}-M\tau_{\theta }$$

where

(6) $$M=\left[\begin{array}{ccc} 0 & -1 & 0\\ 1 & 0 & 0\\ 0 & 0 & 0 \end{array}\right]$$

Likewise, the matrix form of the stress vector can be obtained by combining the constitutive equation and geometric equation:

(7) $$\tau_{r}=\left[D_{11}\right]\frac{\partial u}{\partial r}+\frac{1}{r}\left[D_{12}\right]\left(\frac{\partial u}{\partial \theta }+Mu\right)+\left[D_{13}\right]\frac{\partial u}{\partial z}$$

(8) $$\tau_{\theta }=\left[D_{21}\right]\frac{\partial u}{\partial r}+\frac{1}{r}\left[D_{22}\right]\left(\frac{\partial u}{\partial \theta }+Mu\right)+\left[D_{23}\right]\frac{\partial u}{\partial z}$$

(9) $$\tau_{z}=\left[D_{31}\right]\frac{\partial u}{\partial r}+\frac{1}{r}\left[D_{32}\right]\left(\frac{\partial u}{\partial \theta }+Mu\right)+\left[D_{33}\right]\frac{\partial u}{\partial z}$$

where the **D_ij_** matrix consists of elastic constants. By combining Equations (5)–(9), the guided wave characteristic equation of the semi-infinite domain cladding pipe structure can be derived:

(10) $$\begin{array}{l} -k^{2}\left[D_{22}\right]u+ikr\left(\left[D_{12}\right]+\left[D_{21}\right]\right)\frac{\partial u}{\partial r}+ik\left(\left[D_{22}\right]M+M\left[D_{22}\right]\right)u\\ +r\left(\left[D_{11}\right]+\left[D_{12}\right]M+M\left[D_{21}\right]\right)\frac{\partial u}{\partial r}+r^{2}\left[D_{11}\right]\frac{\partial^{2}u}{\partial r^{2}}+M\left[D_{22}\right]Mu+r^{2}\rho \omega^{2}u=0 \end{array}$$

The detailed derivation of Equation (10) can be found in our previous work [23].

According to the Snell law [24], the acoustic waves in the substratum and cladding have the same harmonic factor $e^{i\left(k\theta -\omega t\right)}$, that is, *k^s^* = *k^c^* = *k*, where the superscript *s* represents the substratum layer and the superscript *c* represents the cladding. Since the SH guided wave propagates in the cladding at a certain angle, an additional wave number *k_r_* is required to represent the partial wave propagating in the *r* direction. The SH wave number vector in the cladding is $k_{c}={\left[\begin{array}{cc} k & k_{r} \end{array}\right]}^{T}$, and the wave number vector |*k_c_*| = *ω*/*c_s_* is determined by the shear wave velocity *c_s_* and the angular frequency *ω* of the cladding. Also, it is assumed that the SH wave in the cladding has an unknown amplitude *U*. In addition, the wave number components in the cladding satisfy the triangular relationship:

(11) $$k_{c}^{2}=k^{2}+k_{r}^{2}$$

Here, the displacement component in the cement cladding (*u^c^*) and steel layer (*u^s^*) can be expressed as follows:

(12) $$u^{c}=Ue^{ik_{r}\left(r-b\right)}e^{i(k\theta -\omega t)}$$

(13) $$u^{s}=u^{s}\left(r\right)e^{i(k\theta -\omega t)}$$

A Legendre orthogonal polynomial expansion of the displacements within the substratum is carried out, the matrix equations are constructed by analyzing the propagation process of the SH guided wave at the interface between the substratum and the semi-infinite domain cladding, and the nonlinear eigenvalue problem is transformed into a polynomial eigenvalue problem by an appropriate transformation of the variables.

To solve the dispersion characteristic equation, Legendre orthogonal polynomials are used to expand the SH guided wave displacement *u^s^* in the steel layer, which can be expressed as follows:

(14) $$u^{s}\left(r\right)=\sum_{n=0}^{N-1}\Psi_{n}Q_{n}(\chi )$$

where $Q_{n}\left(\chi \right)$ is the Legendre polynomial of the *nth* order on the interval $\chi \in \left[-1,1\right]$, $\Psi_{n}$ is the Legendre polynomial expansion coefficient, and *N* is the cut-off order of the Legendre polynomials. Since the Legendre polynomial interval is $\chi \in \left[-1,1\right]$, it is necessary to transform the coordinates *r* (a ≤ *r* ≤ b) to the Legendre polynomial work range by

(15) χ= ℓ(r−R), ℓ=2h

Obviously, *R* = (*a* + *b*)/2 is the mean radius of hollow cylinder. We can have

(16) $$\begin{array}{l} r^{2}={\left(\frac{h}{2}\chi +R\right)}^{2}=\left(\frac{h^{2}}{4}\chi^{2}+R^{2}+hR\chi \right)\\ r^{2}\frac{\partial^{2}u}{\partial r^{2}}=\chi^{2}\frac{\partial^{2}u}{\partial \chi^{2}}+\ell^{2}R^{2}\frac{\partial^{2}u}{\partial \chi^{2}}+\ell^{2}hR\chi \frac{\partial^{2}u}{\partial \chi^{2}}\\ r^{2}\frac{\partial u}{\partial r}=\frac{h}{2}\chi^{2}\frac{\partial u}{\partial \chi }+\ell R^{2}\frac{\partial u}{\partial \chi }+2R\chi \frac{\partial u}{\partial \chi }\;r\frac{\partial u}{\partial r}=\chi \frac{\partial u}{\partial \chi }+\ell R\frac{\partial u}{\partial \chi } \end{array}$$

Substituting Equations (13)–(16) into Equation (10), multiplying the order *m* Legendre polynomials on both sides of the formula, and then integrating *χ* from −1 to 1, yields

(17) $$\begin{array}{l} -k^{2}\left[D_{22}^{s}\right]\sum_{n=0}^{N-1}\int_{-1}^{1}\Psi_{n}Q_{n}(\chi )Q_{m}(\chi )d\chi +ik\left(\left[D_{12}^{s}\right]+\left[D_{21}^{s}\right]\right)\left(\chi +\ell R\right)\sum_{n=0}^{N-1}\int_{-1}^{1}\Psi_{n}\frac{\partial Q_{n}(\chi )}{\partial \chi }Q_{m}(\chi )d\chi \\ +ik\left(\left[D_{22}^{s}\right]M+M\left[D_{22}^{s}\right]\right)\sum_{n=0}^{N-1}\int_{-1}^{1}\Psi_{n}Q_{n}(\chi )Q_{m}(\chi )d\chi +\rho \omega^{2}\left(\;\frac{\chi^{2}}{\ell^{2}}+R^{2}+hR\chi \right)\sum_{n=0}^{N-1}\int_{-1}^{1}\Psi_{n}Q_{n}(\chi )Q_{m}(\chi )d\chi \\ +\left[D_{11}^{s}\right]\left(\chi^{2}+\ell^{2}R^{2}+\ell^{2}hR\chi \right)\sum_{n=0}^{N-1}\int_{-1}^{1}\Psi_{n}\frac{\partial^{2}Q_{n}(\chi )}{\partial \chi^{2}}Q_{m}(\chi )d\chi +M\left[D_{22}^{s}\right]M\sum_{n=0}^{N-1}\int_{-1}^{1}\Psi_{n}Q_{n}(\chi )Q_{m}(\chi )d\chi \\ +\left(\left[D_{11}^{s}\right]+\left[D_{12}^{s}\right]M+M\left[D_{21}^{s}\right]\right)\left(\chi +\ell R\right)\sum_{n=0}^{N-1}\int_{-1}^{1}\Psi_{n}\frac{\partial Q_{n}(\chi )}{\partial \chi }Q_{m}(\chi )d\chi =0 \end{array}$$

where the linear operator expressions $\chi \frac{\partial Q_{n}(\chi )}{\partial \chi }$, $\frac{\partial Q_{n}(\chi )}{\partial \chi }$, $\frac{\partial^{2}Q_{n}(\chi )}{\partial \chi^{2}}$, $\chi \frac{\partial^{2}Q_{n}(\chi )}{\partial \chi^{2}}$, $\chi^{2}\frac{\partial^{2}Q_{n}(\chi )}{\partial \chi^{2}}$, $\chi^{2}Q_{n}(\chi )$, and $\chi Q_{n}(\chi )$ can be found in ref [25].

In addition, for the circumferential SH guided wave in the semi-infinite domain cladding pipe structure, the inner surface of the substratum should satisfy the stress-free boundary condition, and the interface between the substratum and the cladding should satisfy the stress and displacement continuity conditions. The corresponding boundary conditions are shown as follows:

(18) $$\left[D_{11}^{s}\right]\ell \sum_{n=0}^{N-1}\Psi_{n}{\left(-1\right)}^{n}\left(\frac{n\left(n+1\right)}{2}\right)+\frac{1}{a}\left[D_{12}^{s}\right]M\sum_{n=0}^{N-1}\Psi_{n}{\left(-1\right)}^{n}+\frac{1}{a}\left[D_{12}^{s}\right]ik\sum_{n=0}^{N-1}\Psi_{n}{\left(-1\right)}^{n}=0$$

(19) $$\begin{array}{l} \left[D_{11}^{s}\right]\ell \sum_{n=0}^{N-1}\Psi_{n}\left(\frac{n\left(n+1\right)}{2}\right)+\frac{1}{b}\left[D_{12}^{s}\right]M\sum_{n=0}^{N-1}\Psi_{n}+\frac{1}{b}\left[D_{12}^{s}\right]ik\sum_{n=0}^{N-1}\Psi_{n}\\ -ik\frac{1}{b}\left[D_{12}^{c}\right]U-\frac{1}{b}\left[D_{12}^{c}\right]MU-ik_{r}\left[D_{11}^{c}\right]U=0 \end{array}$$

(20) $$\sum_{n=0}^{N-1}\Psi_{n}-U=0$$

By combining Equations (17)–(20), the system of linear equations can be rearranged as follows:

(21) $$\begin{array}{l} F\left(k\right)q=0\\ F\left(k\right)=k^{2}A_{2}+ikA_{1}+A_{0}+ik_{r}A_{r}\\ q_{\left(3N+1\right)\times 1}=\left(\begin{array}{c} {\Omega^{s}}_{3N\times 1}\\ U \end{array}\right)\\ {\Omega^{s}}_{3N\times 1}={{\left[\Psi_{r}^{1},\Psi_{\theta }^{1},\Psi_{z}^{1},\Psi_{r}^{2},\Psi_{\theta }^{2},\Psi_{z}^{2},\ldots ,\Psi_{r}^{N},\Psi_{\theta }^{N},\Psi_{z}^{N}\right]}_{3\times N}}^{T} \end{array}$$

where *A*_2_, *A*_1_, *A*_0_, and *A_r_* are the corresponding coefficient matrices, and *q* is the matrix consisting of the Legendre polynomial expansion coefficients and the unknown amplitude *U*. To solve the above nonlinear eigenvalue problem, it is necessary to transform it into a polynomial eigenvalue problem. Here, the auxiliary variable *γ* is introduced such that the following holds:

(22) $$k=k_{c}\frac{\gamma +\gamma^{-1}}{2}$$

From Equation (11), the corresponding wave number component *k_r_* is given by

(23) $$k_{r}=k_{c}\frac{\gamma -\gamma^{-1}}{2i}$$

By substituting Equations (22) and (23) into Equation (21), and multiplying by 4*γ*^2^, the following polynomial matrix function can be obtained:

(24) $$\begin{array}{l} P\left(\gamma \right)=4\gamma^{2}F\left(k\right)\\ =k_{c}^{2}A_{2}\gamma^{4}+\left(2ik_{c}A_{1}+2k_{c}A_{r}\right)\gamma^{3}+\left(2k_{c}^{2}A_{2}+4A_{0}\right)\gamma^{2}+\left(2ik_{c}A_{1}-2k_{c}A_{r}\right)\gamma +k_{c}^{2}A_{2}\\ =p_{4}\gamma^{4}+p_{3}\gamma^{3}+p_{2}\gamma^{2}+p_{1}\gamma +p_{0} \end{array}$$

Thus, *F*(*k*)q = 0 is converted to *P*(*γ*)q = 0. By introducing $Q={\left[\begin{array}{cccc} \gamma^{3}q & \gamma^{2}q & \gamma q & q \end{array}\right]}^{T}$, the Equation (24) is transformed into a linear eigenvalue problem. The corresponding matrix equations are as follows:

(25) $$\left(R-\gamma S\right)Q=0$$

where $R=\left[\begin{array}{cccc} -p_{3} & -p_{2} & -p_{1} & -p_{0}\\ I & 0 & 0 & 0\\ 0 & I & 0 & 0\\ 0 & 0 & I & 0 \end{array}\right]$, $S=\left[\begin{array}{cccc} p_{4} & 0 & 0 & 0\\ 0 & I & 0 & 0\\ 0 & 0 & I & 0\\ 0 & 0 & 0 & I \end{array}\right]$, and *I* is the unit matrix.

**Figure 2:**
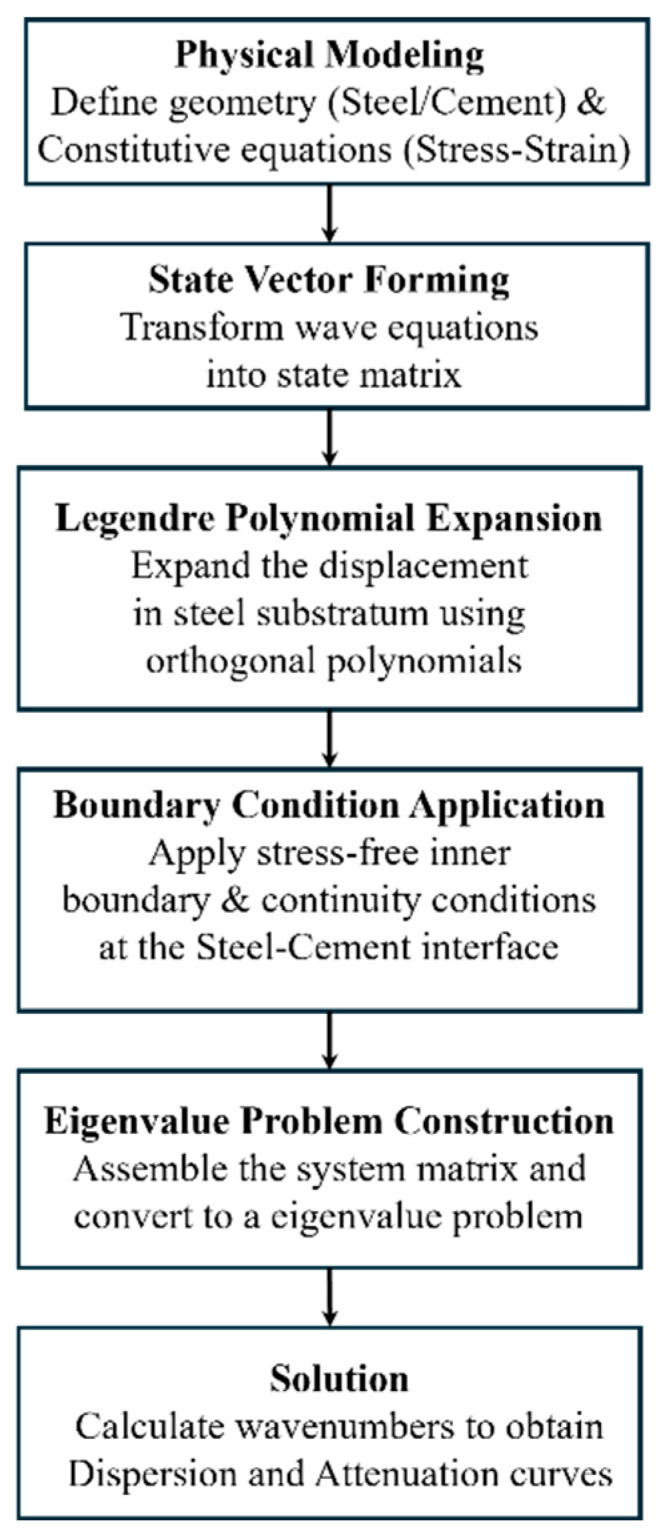
Flowchart of the theoretical modeling process for C-SH guided waves.

The relationship between the complex eigenvalue *γ* and the angular frequency *ω* can be obtained by calculating the eigenvalues of Equation (25). The relationship between the complex wave number *k* and the angular frequency *ω* can be obtained. The phase velocity *c_ph_* = *ω*/Re(*k*) and the dispersion curves can be obtained through the real part of the complex wave number *k*. The attenuation *A_tt_* = 8.686 × Im(*k*) and the attenuation curves can be obtained through the imaginary part of the complex wave number *k*. Meanwhile, we also provide a flowchart to illustrate the modeling steps, which will make the theoretical formulation more accessible, as shown in Figure 2.

### 2.2. Numerical Result and Comparison

**Figure 3:**
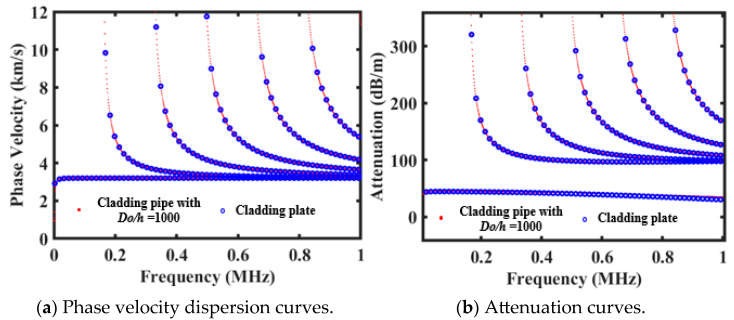
Comparison of theoretical results for cladding pipe (*Do/h* = 1000) and cladding plate.

To verify the feasibility and accuracy of the proposed method, the propagation characteristics of circumferential SH (C-SH) guided waves in cementing casing structures were analyzed numerically. It should be noted that as the ratio of the outer diameter to the thickness of steel pipes gradually increases, the structure of the semi-infinite domain clad pipes progressively approaches that of the corresponding plate structures. Here, the proposed method was employed to calculate the dispersion curves and attenuation curves of C-SH guided waves in steel pipe structures with semi-infinite cement linings. The diameter to thickness ratio is *Do*/*h* = 1000, and thickness *h* is equal to 10 mm. The parameters of the steel pipe and cement material are shown in Table 1. As shown in Figure 3, the numerical results for the cementing casing were compared with the SH wave propagation characteristics of plate structures with equivalent stacking properties. The phase velocity dispersion curve and attenuation curve of SH guided waves in semi-infinite cement-coated plates are obtained from Dispersev2.0.20a software, where the blue circles are the theoretical results of the semi-infinite domain cladding plate, and the red dots are the theoretical results of the semi-infinite domain cladding pipe when the *Do*/*h* = 1000. It can be seen that the calculation results of both the dispersion curves and attenuation curves are in good agreement, which can effectively verify the accuracy of the proposed method.

## 3. Simulation Analysis of C-SH Wave in Cementing Casing

### 3.1. Acoustic Time-Domain Modeling

**Figure 4:**
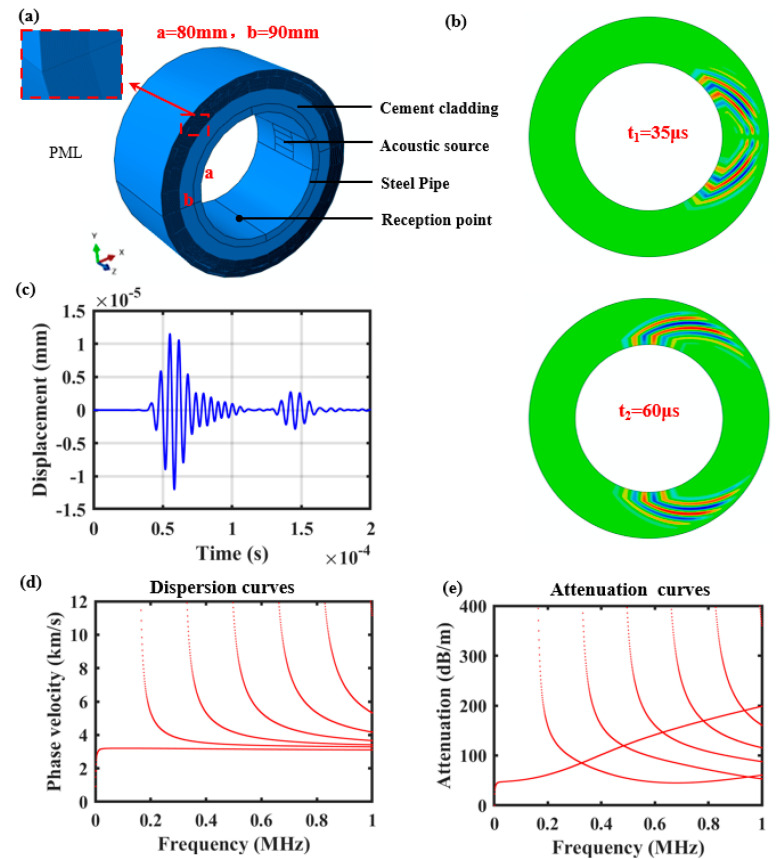
(**a**) Schematic diagram of cemented casing simulation model; (**b**) shows the propagation cloud map of C–guided waves along the cemented casing at 35 μs and 60 μs, respectively; (**c**) illustrates the time–domain waveform at the receiving point; (**d**,**e**) represent the disperse curves and attenuation curves of C–SH guided wave for cemented casing.

To analyze the time-domain propagation process of the C-SH guided wave in the cementing casing, a 3D finite element model of the cementing casing is established using ABAQUSv6.14 software, as shown in Figure 4a. The simulation area mainly consists of two parts, the steel casing and the cement cladding, and the steel and cement properties are consistent with the theoretical model. Likewise, the inner radius of the steel pipe is 80 mm and the thickness is set to 10 mm. Due to the fact that the circumferential SH guided wave propagates only in the circumferential direction, the axial length in the *z*-direction is set to 120 mm to shorten the computation time. A perfect matching layer (PML) is added to the outer wall of the cement cladding to model the semi-infinite domain cement cladding. The damping coefficient of the perfectly matched layer is calculated as follows:

(26) $$a_{n}=a_{\max}\cdot {\left(\frac{n\cdot l}{L}\right)}^{3}$$

where *a_n_* is the damping coefficient of different matching layers, *a*_max_ is the maximum value of the damping coefficient, *l* is the thickness of the matching layer, and *L* is the total thickness of the matching layer. In the perfectly matched layer, the cement material properties are the same between adjacent cement layers and the damping coefficient is varied so that the acoustic energy is attenuated layer by layer. Theoretically, the thinner the thickness of the damping layer is, the better the absorption effect of the PML is, but it will lead to a finer mesh division of the simulation and lower computational efficiency. Under the premise of ensuring the calculation accuracy, the thickness of the damping layer is set to 1 mm and the number of damping layers is 22. While a thinner damping layer theoretically offers better absorption, it requires a finer mesh. Through preliminary convergence testing, we determined that a configuration of 22 layers with a thickness of 1 mm per layer provided the optimal balance, effectively eliminating boundary reflections while maintaining calculation accuracy.

Based on the SH guided wave Lorentz force excitation mechanism, the acoustic source is excited in the form of array loading on the inner wall of the steel pipe. The loading area consists of four rectangles with a length of 40 mm and a width of 10 mm. Apply identical load signals of opposite directions along the z-axis to two adjacent regions. The excitation was applied as surface tractions on the inner wall of the steel pipe. To selectively excite the SH0 mode, which involves shear motion polarized in the axial direction propagating circumferentially, the load was applied in the z-direction. Specifically, identical load signals with opposite directions were applied to adjacent magnet regions. This alternating shear loading pattern matches the periodicity of the SH0 wavelength, thereby maximizing the generation of the desired mode while suppressing other modes. The corresponding signals are five-cycle sine waves modulated by the 160 kHz Hanning window. The mesh size was set to 1 mm, which satisfies the convergence criterion of being significantly smaller than 1/10 of the minimum wavelength. At the excitation frequency of 160 kHz, the theoretical wavelength of the SH0 wave is approximately 20 mm. The reception point is set on the inner wall of the casing in the same plane as the center point of the acoustic source, with the center of the sound source and the reception point separated by 1/4 of the circumference. The simulation solver adopts the power display solver, and in order to ensure the accuracy of the calculation results, the theoretical solving time step should be less than 1/8 of the period, and here the solving time step is set to 0.1 μs. To ensure the accuracy of the simulation results, the grid size should be less than one tenth of the wavelength. Therefore, the mesh is divided into an ortho-hexahedral mesh with 1 mm edge length. The C-SH guided waves vibrate in the z-direction and propagate in the θ-direction, the object of analysis is the z-direction displacement at the receiving point. Shown in Figure 4b are the cloud views of the x–y surface displacement at the moments of t = 35 μs and t = 60 μs. It can be seen that the SH guided wave can be excited effectively and propagated along the θ direction.

In addition, the corresponding C-SH guided wave dispersion curves and attenuation curves of the cementing casing were numerically calculated by using the proposed theoretical method, as shown in Figure 4d,e. Meanwhile, we extracted the *z*-direction displacement signal at the receiving point, as shown in Figure 4c. It can be seen that the first wave packet is a direct wave propagating 1/4 of the circumference, and the second wave packet represents the time-domain signal of acoustic wave propagation over three-quarters of the circumference. By acquiring the time of flight between the 1/4 perimeter direct wave signal and the excitation signal, the wave velocity of the C-SH guided wave is 3072.6 m/s, and the theoretical velocity at *f* = 160 kHz is 3198.7 m/s from Figure 4d, which are in good agreement. The computational results show that the established model generates SH0 mode guided waves.

### 3.2. Bonding Defects and C-SH Guided Wave Attenuation

**Figure 5:**
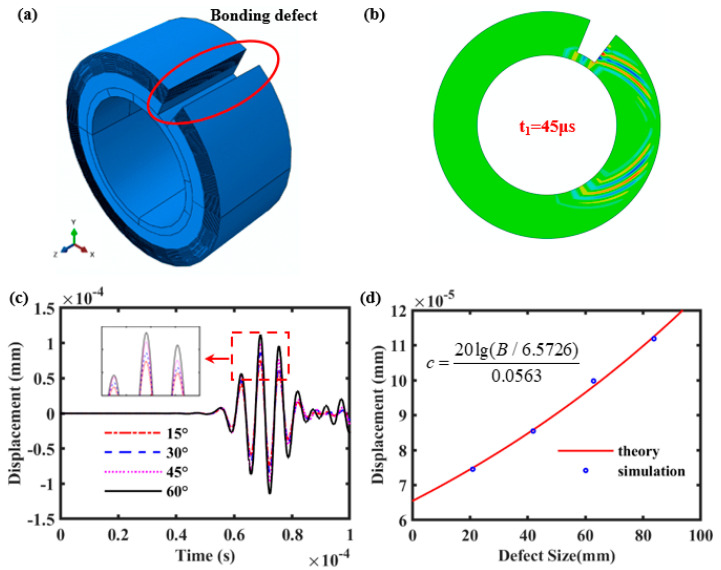
(**a**) Schematic diagram of cemented casing simulation model with bonding defect; (**b**) shows the propagation cloud map of C–SH waves along the cemented casing at 45 μs; (**c**) represents the time–domain waveform for cemented casing with different defect angle; (**d**) illustrates the distribution curve of attenuation characteristic for cemented casing.

To investigate the interaction between bonding defects at the interface of the steel pipe and cement and circumferential SH guided waves, different sizes of bonding defects are set up in the above cementing casing model, as shown in Figure 5a. Also, the location of the receiving point is set to be 1/3 of the circumference from the center of the acoustic source. Here, the bonding defect is located between the acoustic source and the reception point, and the defect completely penetrates the cement cladding in the *z*-direction and in the radial direction. Defects are stepped in the circumferential direction in steps of 15°, gradually increasing from 15° to 60°. The simulation cloud view at *t* = 45 μs displacement is shown in Figure 5b. It can be seen that the C-SH guided wave which propagates along the steel casing will transmit shear waves into the surrounding cement medium. Moreover, when the bonding defect occurs at the steel–cement interface, the SH guided wave will not transmit shear waves at the defect, which results in a higher amplitude of the SH guided wave in the presence of a bonding defect than without defect. Figure 5c shows the *z*-direction displacement signals of bonding defects of different sizes extracted at the reception point. It can be seen that the time of flight of the direct wave for bonding defects of different sizes is almost constant, while the wave amplitude increases with the increase in the defect size.

Meanwhile, the variation in the C-SH guided wave amplitude correlates with the sound field attenuation coefficient. Assuming that the C-SH wave amplitude at the defect-free place is *A*, the leakage attenuation coefficient is defined as *α* (dB/mm), and the corresponding wave amplitude is *B* when there is a *c* mm length defect in the *θ* direction. Based on the attenuation characteristic expression, the relationship between the length of the bonding defect (*c*) and the amplitude (*B*) can be expressed as follows:

(27) $$c=\frac{20\mathrm{lg}(B/A)}{\alpha }$$

To observe the interaction between the size of bonding defects and the C-SH guided waves, the displacement signals of different sizes of bonding defects were compared with the theoretical calculations from Equation (27), as shown in Figure 5d. The blue circles show the displacement of simulated signals and the red solid line shows the curve of the displacement against the size of the bonding defects, which is the relationship between *B* and *c* calculated by Equation (27). As can be seen from Figure 5d, the simulation results match well with the theoretical data. That is to say, the size information of the bonding defects can be obtained from the amplitude information of the C-SH wave, so as to achieve the quantitative characteristics of the bonding defects.

## 4. Cementing Casing Experiment Analysis

**Figure 6:**
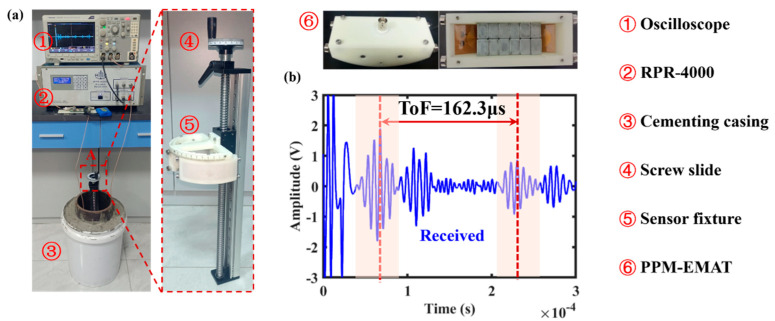
(**a**) Ultrasonic C–SH guided wave experiment system for cemented casing; (**b**) shows the guided wave time–domain signal distribution of cemented casing without defect.

The potential connection between acoustic wave propagation characteristics and the bonding defects utilizing theory and simulation have analyzed. Based on this, the circumferential guided wave detection system is built to acquire the C-SH characteristic parameters for cementing casing, as shown in Figure 6. The thickness of the casing in the cementing casing specimen is 10 mm, the outer diameter is 180 mm, and the height is 400 mm, which is consistent with the theoretical calculation size, and the material used is 45# steel. Also, the cementing casing test specimen uses Grade G cement. The cement slurry was prepared with a standard water-to-cement ratio (0.44) and cured under standard laboratory conditions (25 °C, >90% humidity) for 7 days to ensure stable mechanical properties. The measured density of the cured cement is 1.8 g/cm^3^, and the shear wave velocity is approximately 1700 m/s, consistent with the parameters used in the simulation. The electromagnetic acoustic sensors utilize screw slides and sensor fixtures held in the inner wall of the steel pipe. The 160 kHz 5-cycle sinusoidal pulse signal was burst into the coil of the excitation EMAT by RPR-4000 (RITEC, Inc. Warwick, RI, USA), the conditioning circuit board implements the bandpass filtering and gain of the SH0 signal of the receiving EMAT, and the signal can be observed and acquired by the digital oscilloscope.

The PPM-EMAT primarily consists of permanent magnets, coils, an outer casing, and a top cover. The physical components of the permanent magnets and coils are shown in Figure 6, with the straight section of the coil positioned directly beneath the permanent magnets. The coil is a track-shaped design manufactured via printed flexible circuit boards, comprising four layers with each layer folded back 20 turns. The magnets are neodymium iron boron permanent magnets, totaling 24 pieces arranged in two layers. Each permanent magnet measures 15 mm × 10 mm × 5 mm (length × width × height) and is magnetized along its thickness. Adjacent permanent magnets have opposite magnetic poles, creating a periodic magnetic field. The excitation and reception sensors are separated by 1/3 of the circumference in the circumferential direction. The experimental signals of the defect-free cementing casing are shown in Figure 6b. The propagation distances for bi-directional C-SH guided waves are one-third (first wave packet) and two-thirds (third wave packet) of the circumference, respectively. The corresponding time of flight (TOF) is 162.3 μs, and the experimental wave velocity of the SH0 guided wave is 3097.1 m/s, which is in good agreement with the theoretical as well as the simulated wave velocity.

**Figure 7:**
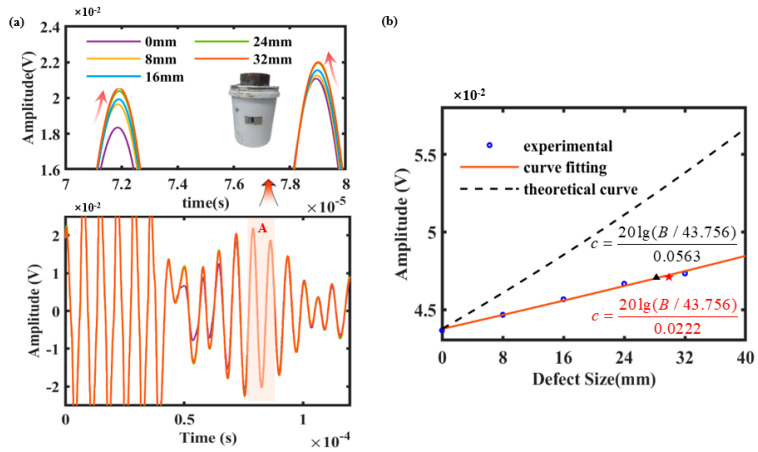
(**a**) Shows the C–SH guided wave time–domain signal distribution of cemented casing with different bonding defect size; (**b**) illustrates the comparison between the fitted experimental decay distribution curve and theoretical data.

Subsequently, experimental analysis was conducted on the defective specimens using the established experimental testing system. It is necessary to prepare cementing casing specimens with bonding defects; here, the bonding defects are of size 40 mm in the axial direction and extend through the entire cement cladding in the radial direction. To explore the interaction between the defect size of circumferential bonding and the C-SH guided waves, the cementing casing with different bonding defects (0 mm, 8 mm, 16 mm, 24 mm, 32 mm) are tested experimentally, as shown in Figure 7a. During the preparation of defective cemented casing specimens, a defect with a predefined axial length of 8 mm was introduced at the cement-to-casing interface and subsequently tested. The defect was then progressively expanded in 8 mm increments. Acoustic transmission data were experimentally measured at defect lengths of 18 mm, 24 mm, and 32 mm to investigate the influence of different defect lengths on circumferential SH waves. Meanwhile, the detailed region A in Figure 7 clearly illustrates the relationship between the wave behavior of C-SH and the defect size. That is to say, it can be seen that the amplitude of the C-SH guided wave increases gradually with the increase in the defect size. This is because no energy leakage along the cement lining occurs in the circumferential SH0 acoustic field at the defect location, resulting in a significant increase in the amplitude of the corresponding guided wave.

To observe the distribution relationship between the size of the bonding defects and the C-SH guided wave, the corresponding peak-to-peak values of the wave signals at different sizes were extracted, as show in the blue circle in Figure 7b, and the red solid line shows the fitted experimental decay distribution curve based on Equation (27). The black dashed line represents the theoretical decay distribution curve from Figure 5d, and the C-SH wave amplitude at the defect-free place is *A* = 43.756 mV; the corresponding attenuation coefficient is defined as *α* = 56.3 dB/m. As can be seen from Figure 7b, the experimental attenuation coefficient is 22.2 dB/m, which is smaller than the theoretical attenuation coefficient. This is mainly because the combined state of the steel pipe and the cement is not as perfect as the simulation model. Meanwhile, the acoustic energy transmitted through the steel pipe into the cement medium is significantly lower than that in the simulated sound field. Theoretical derivations and finite element simulations assume a “perfect bonding” between the steel pipe and cement, where displacements and stresses at the interface are completely continuous. This idealized condition allows ultrasonic energy to “leak” maximally from the steel pipe into the cement layer, causing C-SH guided waves propagating within the pipe to decay extremely rapidly. In practice, however, the cementing interface is often affected by microscopic voids or cement shrinkage during curing, creating a “non-perfect coupling” state. This is analogous to introducing a layer of acoustic impedance mismatch between the two media, impeding the transmission of shear wave energy into the cement side. Consequently, more energy remains confined within the steel pipe, resulting in slower wave amplitude attenuation observed experimentally compared to theoretical predictions. This leads to experimentally calculated attenuation coefficients being smaller than the theoretical values. According to Equation (25), the defect size is proportional to the logarithm of amplitude. This implies that in the ordinary coordinate system, it follows an exponential growth curve. Within a small range, the logarithmic curve may exhibit “quasi-linear” characteristics, but this cannot be extrapolated to imply global linearity.

Fundamentally, the amplitude variation in the C-SH guided wave is governed by the ‘energy leakage’ phenomenon at the steel–cement interface. Since the acoustic impedance of the cement allows for shear wave transmission, a perfect bonding interface acts as a continuous energy drain, allowing the shear motion of the steel to transmit energy into the semi-infinite cement cladding. This results in rapid signal decay (high attenuation) as the wave propagates. However, a bonding defect caused by an air gap acts as a stress-free boundary condition that physically decouples the steel from the cement. This decoupling effectively blocks the transmission of shear waves into the cement at the defect location, trapping the acoustic energy within the steel waveguide. Consequently, the presence of a defect reduces the total energy loss along the propagation path. This explains the experimentally observed trend: as the defect size increases, the cumulative leakage decreases, leading to a significant increase in the received signal amplitude (i.e., a decrease in the effective attenuation).

The experimental distribution law is consistent with the simulation result, indicating the feasibility of the circumferential SH guided wave to evaluate the size of bonding defects. This said, our current attenuation fitting curve captures and quantifies a meaningful relationship between defect size and acoustic behavior. Additionally, we have also fabricated cementing casing containing 30 mm cracks, and acquired the amplitude of the C-SH wave by using the established experimental system, as shown in the position of the five-pointed star in Figure 7b. The corresponding predicted defect size 28.122 mm can be obtained from the experimental attenuation fit curve mentioned, as indicated by the black triangle in Figure 7b, and the corresponding deviation is 6.26%. The main reason is that the amplitude measurements in the experiment are subject to the combined effects of multiple uncertainties. For example, due to the Lorentz force mechanism’s extreme sensitivity to distance, even minute variations in the distance between the sensor and the pipe surface can cause exponential fluctuations in signal amplitude. Although fixed fixtures were employed in the experiment, minor surface irregularities on the pipe may still induce amplitude jitter. This explains the discrete distribution of experimental data points (blue circles) near the fitted curve in Figure 7b. It should be noted that the comparison results indicate that the deviation between the actual defect size of the cementing casing and the predicted value is relatively small, demonstrating that the C-SH guided wave attenuation characteristics possess the capability to characterize bonded defects.

## 5. Conclusions

An alternative non-destructive characterization method for cementing casing bonding defects has been proposed based on the propagation characteristics of circumferential guided waves. Considering the cementing casing structure as a steel substratum semi-infinite domain cement cladding pipe structure, the dispersion characteristic of C-SH guided waves in the cementing casing are acquired based on the state vector and Legendre polynomial method. The accuracy of the proposed theoretical approach is verified by comparing the dispersion curves and attenuation curves of cementing casing with the diameter to thickness ratio *Do*/*h* = 1000 and the plates with stacking structure characteristics, and the two types of data match relatively well. Next, a finite element acoustic field simulation model of the cementing casing structure was established to investigate the relationship between the signal amplitude and the bonding defects. The results indicate that as the size of the bonding defect increases, the amplitude of the C-SH guided wave acoustic field exhibits a gradual increase trend, thereby enabling the derivation of the corresponding attenuation distribution function curve. Finally, an EMAT C-SH guided wave experimental system for cementing casing detection is built to acquire the acoustic information with different bonding defect sizes; the attenuation coefficient in the corresponding experimental attenuation distribution curve is smaller than the result obtained from the simulation. It is worth noting that the distribution trends of both are consistent. Moreover, using the constructed experimental system, C-SH acoustic field information containing bonding defects of arbitrary dimensions is collected and substituted into the attenuation distribution curve to obtain predicted dimensional parameters. The error between the predicted results and the actual defect dimensions is less than 6%. In practical applications, it is necessary to recalculate or calibrate the corresponding attenuation coefficients based on cement of different densities. The proposed method offers a novel research approach for non-destructive quantitative characterization of bonding defects in cementing casing. The demonstrated capability to quantitatively assess bonding quality based on attenuation characteristics, especially in conditions where traditional acoustic impedance logging struggles, provides a foundation for developing advanced downhole logging tools. Furthermore, the non-contact nature of the EMAT methodology utilized here offers promising potential for integrating into real-time inspection systems during cementing operations or for long-term well monitoring campaigns.

## Figures and Tables

**Table 1:** Material parameters of steel and cement.

Materials	*ρ* (g/cm^3^)	*C*_11_ (GPa)	*C*_12_ (GPa)	*C*_44_ (GPa)
Steel	7.9	274.9	113.2	80.8
Cement	1.8	16.2	5.8	5.2

## Data Availability

The original contributions of this study have been included in the article.
